# 

**Published:** 1884-04-20

**Authors:** 


### TABLE OF CONTENTS.

ORIGINAL AND SELECTED ARTICLES:

On Malignant Disease, by H. M. Lawson, M.D., Cuthbert, Ga., 121. Case of Phlegmonous Erysipelas, by T. M. Beaty, Okolona, Miss., 130. Seven Common Surgical Follies, 131. Calcium Sulphide in the Treatment of Diabetes Mellitus, 135. Factors Deciding the Sex of the Offspring, 138. Salicylic Acid in Rheumatism, 139. Cure for Stammering, 140.

ABSTRACTS AND GLEANINGS:

Tongue in Health and Disease; Rule for Reducing Dislocations of the Hip Joint; Intestinal Obstruction Cured by Capillary Entero-Puncture, 141. A New Anæsthetic, Bromoform, 142. The Treatment of Hay Fever, 143. Locality of Malaria, 144. How Typhoid is Spread, 145. Treatment of Nævus; Vaseline Cerate, a Convenient Basis for Ointments Intended for Applications to the Eyelids, 146. Rattlesnake Poisoning Treated by Potassium Permanganate 147. A New Form of Eye Bandage, Horrible Hiccough Cured; Negroes and Musk; Flatulence, 148 Sciatica and Lumbago—Injection of Sulphuric Ether; Ferry-boat Amenities, 149. Prof. S. W. Gross on Bichloride of Mercury Solution vs. Carbolic Acid for Killing Germs; Novel Mode of Bleeding; To Prevent Abscess; Effect of Pilocarpin upon the Hair; Death from the Self-Administration of Chloroform; Asthma, 150.

SCIENTIFIC ITEMS:

Electro-generative Torch or Candle; A Pocket Steam Heater; Electric Headlights, 151. New Process for Making Dynamite; Electricity in the Slaughter House; Rainfall in New Mexico and California, 152.

PRACTICAL NOTES AND FORMULÆ.

To Produce Hilarity; Treatment of Typhoid Fever; German Anti-Spasmodic Mixture, 153; A New Injection for Gonorrhœa; Formulas: Chloride of Zinc in Gonorrhœa, 154.

EDITORIALS AND MISCELLANEOUS.

Editorial Notices; Medical Schools of Louisville, Medical Education, etc., 155. Medical Association of Georgia, 156. Communication, 158. Negro College at Nashville; Books and Pamphlets Received, 159. Receipted; Special Notices, 160.

